# Spironolactone: An Anti-androgenic and Anti-hypertensive Drug That May Provide Protection Against the Novel Coronavirus (SARS-CoV-2) Induced Acute Respiratory Distress Syndrome (ARDS) in COVID-19

**DOI:** 10.3389/fmed.2020.00453

**Published:** 2020-07-28

**Authors:** Flavio A. Cadegiani, Carlos G. Wambier, Andy Goren

**Affiliations:** ^1^Department of Endocrinology, Federal University of São Paulo, São Paulo, Brazil; ^2^Corpometria Institute, Brasília, Brazil; ^3^Department of Dermatology, Warren Alpert Medical School of Brown University, Providence, RI, United States; ^4^Applied Biology Inc., Irvine, CA, United States

**Keywords:** COVID-19, SARS-CoV-2, coronavirus, pandemic, spironolactone, ARDS (acute respiratory distress syndrome), androgen receptor antagonist, TMPRSS2

## Introduction

At the onset of the COVID-19 pandemic, mortality following infection of severe acute respiratory coronavirus (SARS-CoV-2) was thought to be solely associated with aging and pre-existing conditions; however, as the pandemic ensued, several large scale epidemiological observations eluded to additional atypical risk factors, particularly hypertension, obesity, and male gender (1–11).

## SARS-CoV-2: Current Knowledge on the Mechanisms of Action

The peculiarities and complexity of SARS-CoV-2 infection patterns precluded definitive findings regarding the mechanisms of infectivity. Current literature suggests that angiotensin-converting enzyme-2 (ACE2) receptor and transmembrane serine protease 2 (TMPRSS2) are the key for SARS-CoV-2 cell entry (12–19). While ACE2 is the coupling site of the spike protein of SARS-CoV-2, TMPRSS2 facilitates SARS-CoV-2 spikes and ACE2 for viral cell entry. Although ACE2 expression is present diffusely, up to 80% of its expression is located in the type-2 pneumocytes (12, 17), which may explain why COVID-19 is predominantly pulmonary, although SARS-COV-2 may affect any organ and system. TMPRSS2 activity is modulated by androgens, which may justify why males are overrepresented among severe COVID-19 infected patients (20).

Current understanding of SARS-CoV-2 allows the division of COVID-19 into two phases (12–18). In a first, early phase, which corresponds to the period of SARS-CoV-2 cell entry, lung membrane-attached ACE2 expression seems to be positively correlated with virus infectivity, while the balance between circulating ACE2, that could protect from lung infectivity by coupling with SARS-CoV-2 and precluding from the entry into the pneumocytes (13–16), and membrane-attached ACE2, may also be relevant.

In a second phase, represented by the inflammatory and immunological responses to SARS-CoV-2 infection, ACE2 is downregulated due to the entry into cell cytoplasm when coupled with the virus. In opposition to the first phase, in the second phase, lung-attached ACE2 expression may be positively correlated with better clinical outcomes, since ACE2 may limit the cytokine storm that underlies the Acute Respiratory Distress Syndrome (ARDS) in COVID-19, while the balance between proinflammatory angiotensin II–angiotensin receptor type 1 (AT1) axis, and the anti-inflammatory angiotensin 1–7—G-coupled Mas receptor (angiotensin 1–7 receptor) axis may also be crucial for level of severity of the second phase (13, 15–19).

## SARS-CoV-2: The Link Between Mechanisms of Action and Risk Factors

The Renin-Angiotensin-Aldosterone System (RAAS) has been shown to be central in COVID-19, since three of the key modulators of SARS-CoV-2 infectivity–angiotensin 1–7, ACE2, and AT1—belong to the RAAS, in addition to the TMPRSS2 expression (12–19).

Disruption of RAAS and ACE2 expression abnormalities are likely the underlying mechanism that links hypertension and obesity as important risk factors for COVID-19 (21–29). Conversely, TMPRSS2 overexpression in response to exposure to androgens may justify the higher occurrence of COVID-19 complications in males (30–33), which can be reinforced by the fact that males under androgen deprivation therapies such as for prostate cancer may experiment decreased risk for ARDS when compared to age-, sex-, and comorbidities-matched subjects (33).

A pro-thrombotic state, and endothelial, hematological, kidney, hepatic, cardiovascular, gonadal, neurological, and gastrointestinal manifestations in COVID-19 are at least partially mediated by ACE2 and TMPRSS2 expressions (34–60).

In summary, aberrancies in ACE2 expression, unbalance between angiotensin II and angiotensin 1–7 levels, and overexpression of TMPRS22 seem to be key factors for the severity of clinical manifestations in COVID-19.

## Spironolactone as a Candidate Against COVID-19

Drugs that address ACE2, any sight of the RAAS, or TMPRSS2 expression are potential candidates for COVID-19. In this context, the use of old drugs against COVID-19 may present major potential benefits over novel drugs for some reasons, including: (1) The well-established long-term safety profile (2) Extensively described risks and contraindications, which allows to prevent its use when contraindicated and monitor for risks directedly; and (3) The lower cost of old, non-patented drugs allows its massive use in public health systems, when clinically indicated.

These observations combined with our understanding of SARS-CoV-2 molecular mechanism of infectivity lead us to believe that spironolactone is an ideal candidate drug for the prophylactic treatment of SARS-CoV-2.

Spironolactone is a safe and well-tolerated anti-hypertensive and anti-androgenic drug used since 1959, that is effective to maintain normal blood levels (61–63), address heart function, and provide cardio- and renoprotection (64–68).

While spironolactone is a safe and unexpensive option, it may act in multiple sites against COVID-19, including: (1) Favorable patterns of ACE2 expression, including potential increase of circulating ACE2, enhancing its potential protective role in SARS-CoV-2, once plasma ACE2 may couple to SARS-CoV-2 and avoid its entry in the cells (24, 69–74); (2) Downregulation of the androgen-mediated TMPRSS2 due to its antiandrogenic activity (75–77), without the adverse events of male sexual castration; (3) Mitigation of the deleterious effects of obesity on the RAAS, possibly reducing obesity-related COVID-19 complications (78, 79); (4) Direct anti-inflammatory and anti-viral effects that could directly avoid pulmonary complications of COVID-19 (80–90).

Hence, spironolactone meets corresponding epidemiological data, mechanistical plausibility, and sufficient safety profile to become a candidate against COVID-19.

For the proper management of spironolactone during COVID-19, since spironolactone mostly targets the virus entry in the cells, which is the hallmark of the first phase of Covid-19, spironolactone should be preferably started during the earlier stages of the infection, prior to the complications of respiratory manifestations, but could also be employed in the second phase, when the inflammatory and immunologic responses become clinically relevant, due to its anti-inflammatory effects (91).

## Conclusion

Abnormal ACE2 expression, angiotensin II and angiotensin 1–7 imbalance, and TMPRSS2 androgen-mediated overactivity seem to be key regulators of SARS-CoV-2 infectivity, in accordance with epidemiological observations of hypertension, obesity, and male sex as being major risk factors. Since spironolactone is a long used safe drug that exhibits concurrent actions in the modulation of ACE2 expression that could avoid SARS-CoV-2 cell entry, attenuation of the harms caused by the overexpression of angiotensin II-AT-1 axis, discloses anti-androgenic activity that can decrease viral priming through TMPRSS2 activity, and has anti-inflammatory effects in the lungs, spironolactone seems to be a plausible candidate for the prophylactic and early treatment of SARS-CoV-2.

## Author Contributions

FC, CW, and AG developed the underlying theories on the present paper, wrote, and reviewed the manuscript in its final format for submission. All authors contributed to the article and approved the submitted version.

## Conflict of Interest

AG is the CEO of the company Applied Biology Inc (Irvine, CA, USA). CW is part of the medical board of the company Applied Biology Inc (Irvine, CA, USA). The remaining author declares that the research was conducted in the absence of any commercial or financial relationships that could be construed as a potential conflict of interest.

## References

[1] Zhou F Yu T Du R Fan G Liu Y Liu Z Clinical course and risk factors for mortality of adult inpatients with COVID-19 in Wuhan, China: a retrospective cohort study. Lancet. (2020) 395:1054–62. 10.1016/S0140-6736(20)30566-3 32171076 PMC7270627

[2] Lauer SA Grantz KH Bi Q Jones FK Zheng Q Meredith HR. The incubation period of Coronavirus Disease (2019) (COVID-19) from publicly reported confirmed cases: estimation and application. Ann Intern Med. (2020) 172:577–82. 10.7326/M20-0504 32150748 PMC7081172

[3] Wu C Chen X Cai Y Xia J Zhou X Xu S. Risk factors associated with acute respiratory distress syndrome and death in patients with coronavirus disease 2019. Pneumonia in Wuhan, China. JAMA Intern Med. (2020) 180:1–11. 10.1001/jamainternmed.2020.0994 32167524 PMC7070509

[4] Liu W Tao ZW Wang L Yuan ML Liu K Zhou L. Analysis of factors associated with disease outcomes in hospitalized patients with 2019 novel coronavirus disease. Chin Med J. (2020) 133:1032–8. 10.1097/CM9.0000000000000775 32118640 PMC7147279

[5] Fang L Karakiulakis G Roth M. Are patients with hypertension and diabetes mellitus at increased risk for COVID-19 infection? Lancet Respir Med. (2020) 8:e21. 10.1016/S2213-2600(20)30116-8 32171062 PMC7118626

[6] Guan WJ Ni ZY Hu Y Liang WH Ou CQ He JX China medical treatment expert group for Covid-19. Clinical characteristics of coronavirus disease 2019 in China. N Engl J Med. (2020) 382:1708–20. 10.1101/2020.02.06.20020974 32109013 PMC7092819

[7] Hajifathalian K Kumar S Newberry C Shah S Fortune B Krisko T. Obesity is associated with worse outcomes in COVID-19: analysis of Early data from New York City. Obesity. (2020). 10.1002/oby.22923. [Epub ahead of print]. 32470210 PMC7283831

[8] Kalligeros M Shehadeh F Mylona EK Benitez G Beckwith CG Chan PA. Association of obesity with disease severity among patients with coronavirus disease (2019). Obesity. (2020) 28:1200–4. 10.1002/oby.22859 32352637 PMC7267224

[9] Palaiodimos L Kokkinidis DG Li W Karamanis D Ognibene J Arora S. Severe obesity, increasing age and male sex are independently associated with worse in-hospital outcomes, and higher in-hospital mortality, in a cohort of patients with COVID-19 in the Bronx, New York. Metabolism. (2020) 108:154262. 10.1016/j.metabol.2020.154262 32422233 PMC7228874

[10] Goren A McCoy J Wambier CG Vano-Galvan S Shapiro J Dhurat R. What does androgenetic alopecia have to do with COVID-19? An insight into a potential new therapy. Dermatol Ther. (2020) 1:e13365. 10.1111/dth.13365 32237190 PMC7228378

[11] Goren A Vaño-Galván S Wambier CG McCoy J Gomez-Zubiaur A Moreno-Arrones OM. A preliminary observation: male pattern hair loss among hospitalized COVID-19 patients in Spain - a potential clue to the role of androgens in COVID-19 severity. J Cosmet Dermatol. (2020) 19:1545–7. 10.1111/jocd.13443 32301221

[12] Hamming I Timens W Bulthuis MLC Lely AT Navis GJ van Goor H. Tissue distribution of ACE2 protein, the functional receptor for SARS coronavirus: a first step in understanding SARS pathogenesis. J Pathol. (2004) 203:631–7. 10.1002/path.1570 15141377 PMC7167720

[13] Bourgonje AR Abdulle AE Timens W Hillebrands JL Navis GJ Gordijn SJ Angiotensin-converting enzyme 2 (ACE2), SARS-CoV-2 and the pathophysiology of coronavirus disease 2019 (COVID-19). J Pathol. (2020) 251:228–48. 10.1002/path.5471 32418199 PMC7276767

[14] Li Y Zhou W Yang L You R. Physiological and pathological regulation of ACE2, the SARS-CoV-2 receptor. Pharmacol Res. (2020) 157:104833. 10.1016/j.phrs.2020.104833 32302706 PMC7194807

[15] Song J Li Y Huang X Chen Z Li Y Liu C. Systematic analysis of ACE2 and TMPRSS2 expression in salivary glands reveals underlying transmission mechanism caused by SARS-CoV-2. J Med Virol. (2020) 1–11. 10.1002/jmv.26045. [Epub ahead of print]. 32441816 PMC7280739

[16] Imai Y Kuba K Ohto-Nakanishi T Penninger JM. Angiotensin-converting enzyme 2 (ACE2) in disease pathogenesis. Circ J. (2010) 74:405–10. 10.1253/circj.CJ-10-0045 20134095

[17] Kuba K Imai Y Rao S Gao H Guo F Guan B. A crucial role of angiotensin converting enzyme 2 (ACE2) in SARS coronavirus–induced lung injury. Nat Med. (2005) 11:875–9. 10.1038/nm1267 16007097 PMC7095783

[18] Hoffmann M Kleine-Wever H Kruger N Muller M Drotsten C Pholhlmann S The novel coronavirus (2019) (2019-nCoV) uses the SARS coronavirus receptor ACE2 and the cellular protease TMPRSS2 for entry in target cells. Cell. (2020) 181:1–10. 10.1101/2020.01.31.92904232243785

[19] Ortega JT Serrano ML Pujol FH Rangel HR. Role of changes in SARS-CoV-2 spike protein in the interaction with the human ACE2 receptor: an *in silico* analysis. EXCLI J. (2020) 19:410–7. 10.17179/excli2020-1167 32210742 PMC7081066

[20] Wambier CG Goren A. SARS-COV-2 infection is likely to be androgen mediated. J Am Acad Dermatol. (2020) 83:308–9. 10.1016/j.jaad.2020.04.032R 32283245 PMC7151476

[21] Pirola CJ Sookoian S. Estimation of RAAS-Inhibitor effect on the COVID-19 outcome: a meta-analysis. J Infect. (2020). 10.1016/j.jinf.2020.05.052. [Epub ahead of print]. 32474043 PMC7255761

[22] Cariou B Hadjadj S Wargny M Pichelin M Al-Salameh A Allix I. Phenotypic characteristics and prognosis of inpatients with COVID-19 and diabetes: the CORONADO study. Diabetologia. (2020) 63:1500–15. 10.1007/s00125-020-05180-x 32472191 PMC7256180

[23] Groß S Jahn C Cushman S Bär C Thum T. SARS-CoV-2 receptor ACE2-dependent implications on the cardiovascular system: from basic science to clinical implications. J Mol Cell Cardiol. (2020) 144:47–53. 10.1016/j.yjmcc.2020.04.031 32360703 PMC7191280

[24] South AM Diz DI Chappell MC. COVID-19, ACE2, and the cardiovascular consequences. Am J Physiol Heart Circ Physiol. (2020) 318:H1084–90. 10.1152/ajpheart.00217.2020 32228252 PMC7191628

[25] Engeli S Böhnke J Gorzelniak K Janke J Schling P Bader M. Weight loss and the renin-angiotensin-aldosterone system. Hypertension. (2005) 45:356–62. 10.1161/01.HYP.0000154361.47683.d3 15630041

[26] Pinheiro TA Barcala-Jorge AS Andrade JMO Pinheiro TA Ferreira ECN Crespo TS. Obesity and malnutrition similarly alter the renin-angiotensin system and inflammation in mice and human adipose. J Nutr Biochem. (2017) 48:74–82. 10.1016/j.jnutbio.2017.06.008 28779634

[27] Frantz EDC Giori IG Machado MV Magliano DC Freitas FM Andrade MSB. High, but not low, exercise volume shifts the balance of renin-angiotensin system toward ACE2/Mas receptor axis in skeletal muscle in obese rats. Am J Physiol Endocrinol Metab. (2017) 313:E473–82. 10.1152/ajpendo.00078.2017 28679623

[28] Nagase M Fujita T. Mineralocorticoid receptor activation in obesity hypertension. Hypertens Res. (2009) 32:649–57. 10.1038/hr.2009.86 19521418

[29] Jing F Mogi M Horiuchi M. Role of renin-angiotensin-aldosterone system in adipose tissue dysfunction. Mol Cell Endocrinol. (2013) 378:23–8. 10.1016/j.mce.2012.03.005 22465098

[30] Gebhard C Regitz-Zagrosek V Neuhauser HK Morgan R Klein SL. Impact of sex and gender on COVID-19 outcomes in Europe. Biol Sex Differ. (2020) 11:29. 10.1186/s13293-020-00304-9 32450906 PMC7247289

[31] La Vignera S Cannarella R Condorelli RA Torre F Aversa A Calogero AE. Sex-specific SARS-CoV-2 mortality: among hormone-modulated ACE2 expression, risk of venous thromboembolism and hypovitaminosis D. Int J Mol Sci. (2020) 21:2948. 10.3390/ijms21082948 32331343 PMC7215653

[32] Wambier CG Goren A Vaño-Galván S Ramos PM Ossimetha A Nau G. Androgen sensitivity gateway to COVID-19 disease severity. Drug Dev Res. (2020). 10.1002/ddr.21688. [Epub ahead of print]. 32412125 PMC7273095

[33] Montopoli M Zumerle S Vettor R Rugge M Zorzi M Catapano CV. Androgen-deprivation therapies for prostate cancer and risk of infection by SARS-CoV-2: a population-based study (*n* = 4532). Ann Oncol. (2020). 10.1016/j.annonc.2020.04.479. [Epub ahead of print]. 32387456 PMC7202813

[34] Baig AM Khaleeq A Ali U Syeda H. Evidence of the COVID-19 virus targeting the CNS: tissue distribution, host-virus interaction, and proposed neurotropic mechanisms. ACS Chem Neurosci. (2020) 11:995–8. 10.1021/acschemneuro.0c00122 32167747

[35] Li YC Bai WZ Hashikawa T. The neuroinvasive potential of SARS-CoV2 may play a role in the respiratory failure of COVID-19 patients. J Med Virol. (2020) 92:552–5. 10.1002/jmv.25728 32104915 PMC7228394

[36] Conde Cardona G Quintana Pájaro LD Quintero Marzola ID Ramos Villegas Y Moscote Salazar LR. Neurotropism of SARS-CoV 2: mechanisms and manifestations. J Neurol Sci. (2020) 412:116824. 10.1016/j.jns.2020.116824 32299010 PMC7141641

[37] Natoli S Oliveira V Calabresi P Maia LF Pisani A. Does SARS-Cov-2 invade the brain? Translational lessons from animal models. Eur J Neurol. (2020). 10.1111/ene.14277. [Epub ahead of print]. 32333487 PMC7267377

[38] Helms J Kremer S Merdji H Clere-Jehl R Schenck M Kummerlen C Neurologic features in severe SARS-CoV-2 infection. N Engl J Med. (2020) 382:2268–70. 10.1056/NEJMc2008597 32294339 PMC7179967

[39] Lin L Jiang X Zhang Z Huang S Zhang Z Fang Z. Gastrointestinal symptoms of 95 cases with SARS-CoV-2 infection. Gut. (2020) 69:997–1001. 10.1136/gutjnl-2020-321013 32241899

[40] Cheung KS Hung IFN Chan PPY Lung KC Tso E Liu R. Gastrointestinal manifestations of SARS-CoV-2 infection and virus load in fecal samples from a Hong Kong Cohort: systematic review and meta-analysis. Gastroenterology. (2020). 10.1053/j.gastro.2020.03.065. [Epub ahead of print]. 32251668 PMC7194936

[41] Xiao F Tang M Zheng X Liu Y Li X Shan H. Evidence for gastrointestinal infection of SARS-CoV-2. Gastroenterology. (2020) 158:1831–3.e3. 10.1053/j.gastro.2020.02.055 32142773 PMC7130181

[42] Recalcati S. Cutaneous manifestations in COVID-19: a first perspective. J Eur Acad Dermatol Venereol. (2020) 34:e212–3. 10.1111/jdv.16387 32215952

[43] Mungmunpuntipantip R Wiwanitkit V. COVID-19 and cutaneous manifestations. J Eur Acad Dermatol Venereol. (2020) 34:e246. 10.1111/jdv.16483 32294271 PMC7262075

[44] Zhu H Rhee JW Cheng P Waliany S Chang A Witteles RM Cardiovascular complications in patients with COVID-19: consequences of viral toxicities and host immune response. Curr Cardiol Rep. (2020) 22:32 10.1007/s11886-020-01302-4 32318865 PMC7171437

[45] Kim IC Kim JY Kim HA Han S. COVID-19-related myocarditis in a 21-year-old female patient. Eur Heart J. (2020) 41:1859. 10.1093/eurheartj/ehaa288 32282027 PMC7184491

[46] Kochi AN Tagliari AP Forleo GB Fassini GM Tondo C. Cardiac and arrhythmic complications in patients with COVID-19. J Cardiovasc Electrophysiol. (2020) 31:1003–8. 10.1111/jce.14479 32270559 PMC7262150

[47] Zeng JH Liu YX Yuan J Wang FX Wu WB Li JX. First case of COVID-19 complicated with fulminant myocarditis: a case report and insights. Infection. (2020) 10:1–5. 10.1007/s15010-020-01424-5 32277408 PMC7146072

[48] Xu L Liu J Lu M Yang D Zheng X. Liver injury during highly pathogenic human coronavirus infections. Liver Int. (2020) 40:998–1004. 10.1111/liv.14435 32170806 PMC7228361

[49] Lee IC Huo TI Huang YH. Gastrointestinal and liver manifestations in patients with COVID-19. J Chin Med Assoc. (2020) 83:521–3. 10.1097/JCMA.0000000000000319 32243269 PMC7176263

[50] Musa S. Hepatic and gastrointestinal involvement in coronavirus disease (2019) (COVID-19): what do we know till now? Arab J Gastroenterol. (2020) 21:3–8. 10.1016/j.ajg.2020.03.002 32253172 PMC7174834

[51] Fanelli V Fiorentino M Cantaluppi V Gesualdo L Stallone G Ronco C. Acute kidney injury in SARS-CoV-2 infected patients. Crit Care. (2020) 24:155. 10.1186/s13054-020-02872-z 32299479 PMC7161433

[52] Chu KH Tsang WK Tang CS Lam MF Lai FM To KF. Acute renal impairment in coronavirus-associated severe acute respiratory syndrome. Kidney Int. (2005) 67:698–705. 10.1111/j.1523-1755.2005.67130.x 15673319 PMC7112337

[53] Abobaker A Raba AA. Does COVID-19 affect male fertility? World J Urol. (2020) 21:1–2. 10.1007/s00345-020-03208-w 32318855 PMC7171435

[54] Cardona Maya WD Du Plessis SS Velilla PA. SARS-CoV-2 and the testis: similarity with other viruses and routes of infection. Reprod Biomed Online. (2020) 40:763–4. 10.1016/j.rbmo.2020.04.009 32362571 PMC7162782

[55] Klok FA Kruip MJHA van der Meer NJM Arbous MS Gommers DAMPJ Kant KM Incidence of thrombotic complications in critically ill ICU patients with COVID-19. Thromb Res. (2020) 191:145–7. 10.1016/j.thromres.2020.04.013 32291094 PMC7146714

[56] Llitjos JF Leclerc M Chochois C Monsallier JM Ramakers M Auvray M. High incidence of venous thromboembolic events in anticoagulated severe COVID-19 patients. J Thromb Haemost. (2020) 18:1743–6. 10.1111/jth.14869 32320517 PMC7264774

[57] Panigada M Bottino N Tagliabue P Grasselli G Novembrino C Chantarangkul V. Hypercoagulability of COVID-19 patients in intensive care unit: a report of thromboelastography findings and other parameters of hemostasis. J Thromb Haemost. (2020) 18:1738–42. 10.1111/jth.14850 32302438 PMC9906150

[58] Cui S Chen S Li X Liu S Wang F. Prevalence of venous thromboembolism in patients with severe novel coronavirus pneumonia. J Thromb Haemost. (2020) 18:1421–4. 10.1111/jth.14830 32271988 PMC7262324

[59] Rotzinger DC Beigelman-Aubry C von Garnier C Qanadli SD. Pulmonary embolism in patients with COVID-19: time to change the paradigm of computed tomography. Thromb Res. (2020) 190:58–9. 10.1016/j.thromres.2020.04.011 32302782 PMC7151364

[60] Giannis D Ziogas IA Gianni P. Coagulation disorders in coronavirus infected patients: COVID-19, SARS-CoV-1, MERS-CoV and lessons from the past. J Clin Virol. (2020) 127:104362. 10.1016/j.jcv.2020.104362 32305883 PMC7195278

[61] Georgianos PI Vaios V Eleftheriadis T Zebekakis P Liakopoulos V. Mineralocorticoid antagonists in ESRD: an overview of clinical trial evidence. Curr Vasc Pharmacol. (2017) 15:599–606. 10.2174/1570161115666170201113817 28155610

[62] Hermidorff MM Faria Gde O Amâncio Gde C de Assis LV Isoldi MC. Non-genomic effects of spironolactone and eplerenone in cardiomyocytes of neonatal Wistar rats: do they evoke cardioprotective pathways? Biochem Cell Biol. (2015) 93:83–93. 10.1139/bcb-2014-0110 25488178

[63] Cadegiani FA. Can spironolactone be used to prevent COVID-19-induced acute respiratory distress syndrome in patients with hypertension? Am J Physiol Endocrinol Metab. (2020) 318:E587–8. 10.1152/ajpendo.00136.2020 32297520 PMC7191632

[64] Nakano S Kobayashi N Yoshida K Ohno T Matsuoka H. Cardioprotective mechanisms of spironolactone associated with the angiotensin-converting enzyme/epidermal growth factor receptor/extracellular signal-regulated kinases, NAD(P)H oxidase/lectin-like oxidized low-density lipoprotein receptor-1, and Rho-kinase pathways in aldosterone/salt-induced hypertensive rats. Hypertens Res. (2005) 28:925–36. 10.1291/hypres.28.925 16555582

[65] Dieterich HA Wendt C Saborowski F. Cardioprotection by aldosterone receptor antagonism in heart failure. Part I. The role of aldosterone in heart failure. Fiziol Cheloveka. (2005) 31:97–105. 10.1007/s10747-005-0119-8 16366159

[66] Taira M Toba H Murakami M Iga I Serizawa R Murata S. Spironolactone exhibits direct renoprotective effects and inhibits renal renin-angiotensin-aldosterone system in diabetic rats. Eur J Pharmacol. (2008) 589:264–71. 10.1016/j.ejphar.2008.06.019 18582458

[67] Schjoedt KJ. The renin-angiotensin-aldosterone system and its blockade in diabetic nephropathy: main focus on the role of aldosterone. Dan Med Bull. (2011) 58:B4265. 21466768

[68] Kong EL Zhang JM An N Tao Y Yu WF Wu FX. Spironolactone rescues renal dysfunction in obstructive jaundice rats by upregulating ACE2 expression. J Cell Commun Signal. (2019) 13:17–26. 10.1007/s12079-018-0466-2 29882088 PMC6381374

[69] Takeda Y Zhu A Yoneda T Usukura M Takata H Yamagishi M. Effects of aldosterone and angiotensin II receptor blockade on cardiac angiotensinogen and angiotensin-converting enzyme 2 expression in Dahl salt-sensitive hypertensive rats. Am J Hypertens. (2007) 20:1119–24. 10.1016/j.amjhyper.2007.05.008 17903697

[70] Zhu A Yoneda T Demura M Karashima S Usukura M Yamagishi M. Effect of mineralocorticoid receptor blockade on the renal renin-angiotensin system in Dahl salt-sensitive hypertensive rats. J Hypertens. (2009) 27:800–5. 10.1097/HJH.0b013e328325d861 19516179

[71] Keidar S Gamliel-Lazarovich A Kaplan M Pavlotzky E Hamoud S Hayek T. Mineralocorticoid receptor blocker increases angiotensin-converting enzyme 2 activity in congestive heart failure patients. Circ Res. (2005) 97:946–53. 10.1161/01.RES.0000187500.24964.7A 16179584

[72] Te Riet L van Esch JH Roks AJ van den Meiracker AH Danser AH. Hypertension: renin-angiotensin-aldosterone system alterations. Circ Res. (2015) 116:960–75. 10.1161/CIRCRESAHA.116.303587 25767283

[73] Hamming I Cooper ME Haagmans BL Hooper NM Korstanje R Osterhaus AD. The emerging role of ACE2 in physiology and disease. J Pathol. (2007) 212:1–11. 10.1002/path.2162 17464936 PMC7167724

[74] Patel S Rauf A Khan H Abu-Izneid T. Renin-angiotensin-aldosterone (RAAS): the ubiquitous system for homeostasis and pathologies. Biomed Pharmacother. (2017) 94:317–25. 10.1016/j.biopha.2017.07.091 28772209

[75] Sert M Tetiker T Kirim S. Comparison of the efficiency of anti-androgenic regimens consisting of spironolactone, Diane 35, and cyproterone acetate in hirsutism. Acta Med Okayama. (2003) 57:73–6. 10.18926/AMO/32820 12866746

[76] Steelman SL Brooks JR Morgan ER Patanelli DJ. Anti-androgenic activity of spironolactone. Steroids. (1969) 14:449–50. 10.1016/S0039-128X(69)80007-3 5344274

[77] Broulik PD Stárka L. Antiandrogenic and antirenotropic effect of spironolactone. Endokrinologie. (1976) 68:35–9. 1001267

[78] Vecchiola A Fuentes CA Solar I Lagos CF Opazo MC Muñoz-Durango N. Eplerenone implantation improved adipose dysfunction averting RAAS activation and cell division. Front Endocrinol. (2020) 11:223. 10.3389/fendo.2020.00223 32373073 PMC7186315

[79] Feraco A Armani A Mammi C Fabbri A Rosano GM Caprio M. Role of mineralocorticoid receptor and renin-angiotensin-aldosterone system in adipocyte dysfunction and obesity. J Steroid Biochem Mol Biol. (2013) 137:99–106. 10.1016/j.jsbmb.2013.02.012 23454117

[80] Ji WJ Ma YQ Zhang X Zhang L Zhang YD Su CC. Inflammatory monocyte/macrophage modulation by liposome-entrapped spironolactone ameliorates acute lung injury in mice. Nanomedicine. (2016) 11:1393–406. 10.2217/nnm-2016-0006 27221077 PMC5561989

[81] Ji WJ Ma YQ Zhou X Zhang YD Lu RY Guo ZZ. Spironolactone attenuates bleomycin-induced pulmonary injury partially via modulating mononuclear phagocyte phenotype switching in circulating and alveolar compartments. PLoS ONE. (2013) 8:e81090. 10.1371/journal.pone.0081090 24260540 PMC3834272

[82] Rafatian N Westcott KV White RA Leenen FH. Cardiac macrophages and apoptosis after myocardial infarction: effects of central MR blockade. Am J Physiol Regul Integr Comp Physiol. (2014) 307:R879–87. 10.1152/ajpregu.00075.2014 25100076

[83] Zhang L Hao JB Ren LS Ding JL Hao LR. The aldosterone receptor antagonist spironolactone prevents peritoneal inflammation and fibrosis. Lab Invest. (2014) 94:839–50. 10.1038/labinvest.2014.69 24862968

[84] Ozacmak HS Ozacmak VH Barut F Arasli M Ucan BH. Pretreatment with mineralocorticoid receptor blocker reduces intestinal injury induced by ischemia and reperfusion: involvement of inhibition of inflammatory response, oxidative stress, nuclear factor κB, and inducible nitric oxide synthase. J Surg Res. (2014) 191:350–61. 10.1016/j.jss.2014.04.040 24862878

[85] Kato Y Kamiya H Koide N Odkhuu E Komatsu T Dagvadorj J. Spironolactone inhibits production of proinflammatory mediators in response to lipopolysaccharide via inactivation of nuclear factor-κB. Immunopharmacol Immunotoxicol. (2014) 36:237–41. 10.3109/08923973.2014.921690 24852317

[86] Lieber GB Fernandez X Mingo GG Jia Y Caniga M Gil MA. Mineralocorticoid receptor antagonists attenuate pulmonary inflammation and bleomycin-evoked fibrosis in rodent models. Eur J Pharmacol. (2013) 718:290–8. 10.1016/j.ejphar.2013.08.019 24012780

[87] Fraccarollo D Galuppo P Schraut S Kneitz S van Rooijen N Ertl G. Immediate mineralocorticoid receptor blockade improves myocardial infarct healing by modulation of the inflammatory response. Hypertension. (2008) 51:905–14. 10.1161/HYPERTENSIONAHA.107.100941 18299485

[88] Lacombe B Morel M Margottin-Goguet F Ramirez BC. Specific inhibition of HIV infection by the action of spironolactone in T cells. J Virol. (2016) 90:10972–80. 10.1128/JVI.01722-16 27681137 PMC5110165

[89] Verma D Thompson J Swaminathan S. Spironolactone blocks Epstein-Barr virus production by inhibiting EBV SM protein function. Proc Natl Acad Sci USA. (2016) 113:3609–14. 10.1073/pnas.1523686113 26976570 PMC4822607

[90] Yartaş Dumanli G Dilken O Ürkmez S. Use of spironolactone in SARS-CoV-2 ARDS patients. Turk J Anaesthesiol Reanim. (2020) 48:254–55. 10.5152/TJAR.2020.569 32551456 PMC7279869

[91] Alifano M Alifano P Forgez P Iannelli A. Renin-angiotensin system at the heart of COVID-19 pandemic. Biochimie. (2020) 174:30. 10.1016/j.biochi.2020.04.008 32305506 PMC7161528

